# ALEX versus ISAC multiplex array in analyzing food allergy in atopic children

**DOI:** 10.1186/s12948-022-00177-w

**Published:** 2022-08-27

**Authors:** Laura J. H. Sonneveld, Joyce A. M. Emons, Nicolette J. T. Arends, Lonneke J. Landzaat, Sharon Veenbergen, Marco W. J. Schreurs

**Affiliations:** 1 Department of Pediatrics, Division of Respiratory Medicine and Allergology, Sophia Children Hospital, Erasmus MC University Medical Centre, Dr. Molewaterplein 40, 3015 GD Rotterdam, The Netherlands; 2 Department of Immunology, Laboratory Medical Immunology, Erasmus MC University Medical Centre, Rotterdam, The Netherlands

**Keywords:** Multiplex allergy array, Molecular allergy diagnostics, ALEX, ISAC, Pediatric allergy, Food allergy

## Abstract

ALEX multiplex array is a relatively new multiplex allergy test which analyses more than 120 allergen extracts and 170 molecular components. ISAC is the most used and studied multiplex array to date, offering 112 molecular components. In ten atopic children with multiple food allergies good agreement was observed between ALEX and ISAC sIgE results for nearly all shared food components. Presence of larger number of allergens in ALEX could help clinicians to improve personalized dietary advice. However more positive sensitizations with unknown clinical relevance were found by ALEX, potentially increasing clinical complexity. Pediatric allergists should be aware of this, especially in young atopic children with (severe) eczema who have not introduced all sorts of food yet.

To the editor,

Recently a new multiplex array in molecular allergy diagnostics, ALEX (Allergy Explorer, Macro Array Diagnostics, Vienna, Austria), was launched and became commercially available in 2019. ISAC (Immuno Solic-phase Allergen Chip, Thermo Fisher Scientific, Uppsala, Sweden, available since 2008) is the most frequently used and studied multiplex array to date. ALEX multiplex array represents an extended platform which offers 120 allergen extracts and 170 molecular components (ALEX2) of inhalation, food, animal, latex and insect allergen sources. ISAC multiplex array offers 112 molecular components. ALEX and ISAC share 102 common inhalation- and food-derived allergen components. Previous studies [1–4] showed good agreement between ALEX and ISAC, however none of these studies related both their sensitization results directly to clinical relevant allergies, neither discussed the impact on clinical decision making in children with food allergy.

Analyzing multiple molecular components is considered a third-level diagnostic tool, after Skin Prick Test (SPT) and singleplex specific IgE (sIgE) test for whole allergens extracts. When comparing both platforms it is important to know which allergens are present in both multiplex arrays and which are not (see Table 1 legends). Multiplex tools help clinicians in allergy diagnosis precision medicine to distinguish between clinically relevant [e.g. storage proteins (SP)] and less relevant sensitizations [e.g. cross reacting PR-10 proteins (PR10)]. Both ALEX and ISAC are solid-phase immunoassays which enable the detection of sIgE to multiple individual allergen components. When comparing these multiplex arrays one should be aware that both platforms employ different methodologies and units of measurement [5], and in addition ALEX uses a CCD inhibitor while ISAC does not [1–3, 5]. ALEX reports its results as kUA/L, similar as ImmunoCAP, classified in five different categories (Fig. 1). ISAC results are reported as semi-quantitative ISU-E units classified in four different categories (Fig. 1). A different range is used; 0.3–50 kUA/L for ALEX compared to 0.3–200 ISU-E for ISAC. Hoang et.al [5]. demonstrated in children with food allergen sensitizations that quantitative conversion between different sIgE platforms [ALEX (Macro Array Diagnostics), ISAC present in MeDALL-chip (Thermo Fisher) and EUROLINE (EUROIMMUN)] is possible.

**Table 1:** Patient characteristics

Patient (Sex M/F; age yr)	Food allergies (clinically relevant)	Inhalation allergies (clinically relevant)	Asthma	Eczema	Unexplained anaphylaxis in history	Possibly relevant extra results (negative and positive sensitizations) found by ALEX not present in ISAC	Sensitizations found by ALEX with unknown clinical relevance	*Dietary modifications* or extra challenge tests performed based on ALEX results
1 (M; 16)	Egg, peanut, nuts, coconut, legumes, wheat, seeds, carrot, banana, kiwi	Mites, trees, multiple animals	Yes	Yes	No	Highly positive; all sorts of nuts, legumes (except green bean), cereals incl. buckwheat and seeds Moderately positive: caraway, cricket, storage mite(Lep d 2) Negative: banana extract	Cricket, mealworm (low), caraway, fenugreek, poppy seeds, storage mites, lupin	*No dietary modifications* Optional: challenge test banana or green bean in future
2 (M; 12)	Nuts	Trees, grass, multiple animals	No	No	Yes	Highly positive: all sorts nuts and seeds (except fenugreek) Moderate- highly positive: LTP proteins Act d 10 (kiwi), Mal d 3 (apple), Vit v 1 (grapes), Api g 2 (celery), Zea m 14 (corn). Buckwheat, chickpea extract, storage mites (Gly d 2, Lep d 2)	Storage mites ***Cor a 14 (hazelnut)*** ***Fag e 2 (buckwheat) (both negative in ISAC)***	*No dietary modifications* Food challenge tests performed for poppy seeds and buckwheat: both negative
3 (M;12)	Cow’s milk, nuts, certain legumes (except brown and green beans)	Mites, trees, grass, dog	Yes	No	Yes	Moderately positive: all legumes (except green bean), sunflower and poppy seeds, mustard, beef and pork Negative: extracts and components for pistachio- and macadamia nuts, components cashewnut (Ana o 2, Ana o 3)	Cricket, grasshopper, mealworm and buckwheat (low sensitizations), lupin, seeds incl. poppy seeds and fenugreek	*No dietary modifications* Optional: challenge test cashewnut / pistachio and macadamia nut in future
4 (M: 15)	Nuts, sesame, bean sprouts, legumes (except brown and green beans), kiwi	Mites	Yes	Yes	No	Highly positive; all sorts of nuts, seeds incl. sesame and poppy seeds, buckwheat Moderately positive: caraway, anise, chickpeas Negative/very low: peas, lentils and green bean	Caraway, anise, buckwheat, poppy seeds	*No dietary modifications* Optional: challenge test peas or lentils in future
5 (M; 7)	Cow’s milk, nuts, kiwi, fish *Also avoids seafood*	No clinical relevant	Yes	Yes	No	Highly positive; all sorts of nuts (except macadamia), all sorts of fish (parvalbumins), beef potato and mustard, storage mites (Aca s, Gly d 2, Lep d 2, Tyr p) Moderately positive: chickpeas, lentils, peas, buckwheat, lupin, sunflower, poppy and sesame seeds, beef, pork and rabbit Negative/very low: macadamia nut, all seafood species	Chickpeas, lentils, seeds incl. fenugreek, poppy seeds, mustard, buckwheat, lupin, rabbit meat, storage mites ***Jug r 1 (walnut; negative in ISAC)***	*Introducing seafood allowed* Optional: challenge test macadamia nut in future
6 (M; 13)	Peanut, nuts (except almond)	Mites, trees, grass, multiple animals	Yes	Yes	No	Highly positive; cashewnut, pistachio, pecan- and walnut Moderately positive: pear, storage mites (Gly d 2, Lep d 2) Negative: macadamia- and brazil nut and almond	Storage mites	*No dietary modifications* Optional: challenge test brazil or macadamia nut in future
7 (F; 13)	Cow’s milk, egg, soy, hazelnut *Avoids all nuts and fish*	Mites, trees, grass, multiple animals	Yes	Yes	No	Moderate positive; tuna, carper and sword fish (parvalbumins), north sea shrimp (Troponin C), oyster, cashewnut and brazil nut extract (components negative), caraway and anise, cricket, grasshopper, mealworm, storage mites (Aca s, Tyr p, Lep d 2) Negative/very low: pecan- and walnut, macadamia and pistachio nut	North sea shrimp, fish, cricket, grasshopper, mealworm, storage mites, poppy seeds	*No dietary modifications* Optional: challenge test for any nut in future
8 (M; 11)	Peanut, peas, white beans, bean sprouts, walnut and pecan nut, fish *Also avoids seafood and big amounts of lentils and peas*	Mites, trees, grass, multiple animals	Yes	Yes	No	Highly positive: All nuts (except almond and macadamia), all legumes, all fishes, cricket, grasshopper mealworm, pork, lamb, all storage mites Moderately positive: buckwheat, lupin, corn, figue, strawberry, celery, pumpkin seeds, poppy seeds and fenugreek Negative or very low: seafood	Buckwheat, lupin, pork, cricket, grasshopper, mealworm, storage mites, fenugreek, poppy seeds	*Introducing seafood allowed* *Avoiding insects from diet* Already eating all nuts (except walnut and pecannut) without symptoms
9 (F; 11)	Cashew nut, pistachio nut, kiwi	Mites, trees, grass, cat	No	Yes	No	Highly positive: cashewnut and pistachio nut, north sea shrimp (Troponin C), storage mite (Gly d 2) Moderately positive: cricket and grasshopper, storage mites (Tyr p; Lep d 2, Aca s) Negative: all other nuts	Cricket, grasshopper, storage mites	*No dietary modifications*
10 (M; 8)	Peanut, nuts (except almond), sesame, kiwi	Mites, trees, grass, multiple animals	No	Yes	Yes	Highly positive: all nuts (incl almond), buckwheat, sunflowers seeds, poppy seeds, tuna fish and sword fish (parvalbumines) and chickpeas, storage mites (Lep d2) Moderately positive: lentils, lupin	Chickpeas, lentils, buckwheat, lupin, sunflower and poppy seeds, fish, storage mites	*No dietary modifications* Eating almonds without symptoms

*Extra food allergen components and whole allergen extracts in ALEX, not present in ISAC*

Egg, cow’s milk and peanut: whole allergen extract for egg yolk, egg white and cow’s milk; Gal d 4 (egg white; Lysozyme C) and Ara h 15 (peanut; Oleosine)

Nuts: whole allergen extracts for cashew, brazil, pecan, macadamia and almond; Cor a 11 (hazelnut; 7/8S Globulin); Jug r 2 (walnut; 7/8S Globulin), Jug r 4 (walnut; 11S Globulin), Jug r 6 (walnut; 7/8S Globulin); Mac i 2S (macadamia nut; 2S Albumin); Pis v 1 (pistachio; 2S Albumin), Pis v 2 (pistachio; 11S Globulin subunit) and Pis v 3 (pistachio; 7/8S Globulin)

Legumes: whole allergen extracts for peas, chick peas, lentils and green bean and Gly m 8 (soy; 2S Albumin)

Cereals: oats, quinoa, buckwheat, barley, millet, lupine, rye, spelt, rice and corn extracts and Zea m 14 (corn; nsLTP)

Fish and seafood: whole allergen extracts and components for eight fish species (codfish, carp, herring, mackerel, salmon, stingray, swordfish and tuna), three prawn species (north sea shrimp, pacific white shrimp and northern pink shrimp), crab and lobster and five mollusks species (clam, mussel, oyster, scallop and squid)

Fruits: Whole allergen extracts for: papaya, orange, mango, banana, cherry, pear, blueberry. Act d 10 (kiwi; nsLTP), Cuc m 2 (melon; profillin), Fra a1 + 3 (strawberry; PR10 + nsLTP), Mal d 2 (apple; TLP), Mal d 3 (apple; nsLTP) and Vit v 1 (grape; nsLTP)

Vegetables: whole allergen extracts for: onion, garlic, carrot, avocado, potato and tomato. Api g 2 and Api g 6 (celery; nsLTPs), Dau c 1 (carrot; PR-10), Sola l 6 (tomato; nsLTP)

Seeds: whole allergen extracts for pumpkin seeds, sunflower seeds, poppy seeds, sesame seeds and fenugreek

Spices: whole allergen extracts for paprika, caraway, oregano, parsley, anise and mustard and Sin a 1 (mustard; 2S Albumin)

Meat: whole allergen extracts for beef, chicken, pork, turkey, rabbit, lamb, horse, cricket, grasshopper and mealworm and Sus d 1 (pork; Serumalbumin)

Other animal products:: whole allergen extracts for goat’s milk, camel’s milk, horse’s milk, sheep’s milk

Storage mites: whole allergen extract for Acarus siro (Aca s), Tyrophagus putrescentiae (Tyr p); Blo t 10 (Blomia tropicalis; Tropomyosin), Blo t 21 (Blomia tropicalis), Gly d 2 (Glycyphagus domesticus; NPC2 family), Lep d 2 (Lepidoglyphus destructor, NPC2 family), Tyr p 2 (Tyrophagus putrescentiae; NPC2 family)

*Extra food allergen components in ISAC not present in ALEX*

Cow’s milk: Bos d lactoferrin (cow’s milk; Transferrin)

Fruit: Pru p 1 (peach; PR-10), Act d 8 (kiwi; PR-10)

Meat: Alpha-Gal (Gal-alfa-1,3-gal)

Different results between ALEX and ISAC for overlapping components are highlighted in bold italics

**Fig. 1:**
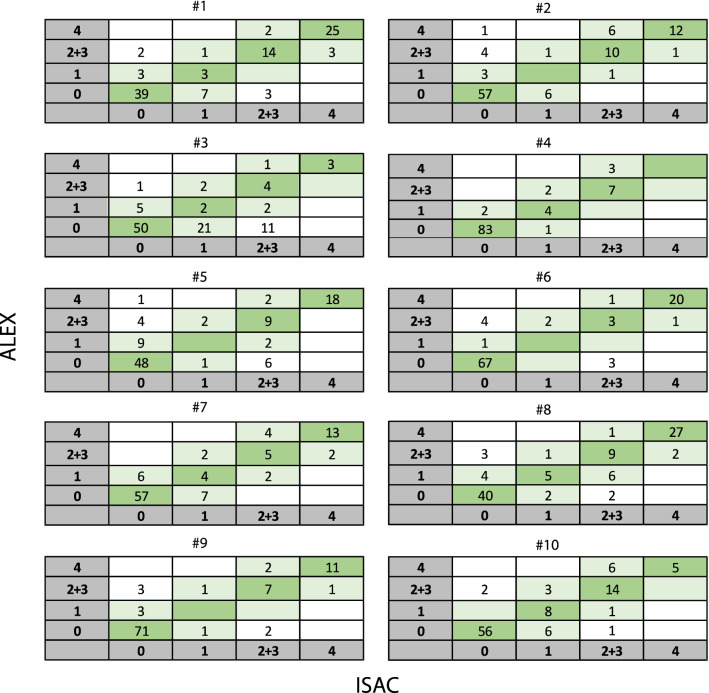
Agreement of ALEX versus ISAC test results by classes defined for ISAC in 10 patients (for all overlapping allergen components) # patient number. ALEX results are classified in five different categories: negative (< 0.3 kUA/L), low (0.3–1 kUA/L), moderate (1–5 kUA/L), high (5–15 kUA/L) and very high (> 15 kUA/L). ISAC results are classified in four different categories: negative (< 0.3 ISU-E), low (0.3–1.0 ISU-E), moderate-high (1–15 ISU-E) and very high > 15 ISU-E). For overall class agreement between ALEX and ISAC results were classified in four sensitization categories [< 0.3 (0) (negative), 0.3–1 (1) (low), 1–15 (2 + 3) (moderate-high), > 15 (4) (very high)]

Our aim was to compare the clinical application of ALEX and ISAC multiplex array in atopic children with multiple food allergies by analyzing:

1. **Overall agreement and differences** for the overlapping allergen components in ALEX and ISAC.
2. **Additional positive sensitizations** found by ALEX (not present in ISAC) and their impact on allergy diagnosis and clinical decision making (dietary modifications / challenge tests).
3. **Negative or very low sensitizations** found by ALEX (not present in ISAC) with clinical implications on dietary advice (introduction of certain foods).

Therefore a pilot study was performed testing ALEX in serum samples of ten children with multiple food allergies who were previously (last year) tested by ISAC. Ten patients with multiple food allergies were included with a mean age of 11,8 years (range 7–16) and the majority was male. Most of them had a diagnosis of eczema (n = 8), asthma (n = 7) and/or allergic rhinitis (n = 9), all requiring medication (Table 1). Our study included children with food allergies for egg (n = 2), cow’s milk (n = 3), peanut (n = 4), nuts (n = 10), legumes (n = 4), soy (n = 1), wheat (n = 1), fish (n = 2), certain fruits/vegetables (n = 5) and seeds (n = 2) (Table 1).

*The overall agreement* between ALEX and ISAC was good with 86.2% agreement (both positive/both negative sensitization) for all shared inhalation- and food allergen components between ALEX and ISAC (n = 102) with a negative percent agreement (NPA, both negative) of 90.3% and a positive percent agreement (PPA, both positive) of 79.5%. Overall class agreement between ALEX and ISAC based on four sensitization categories [< 0.3 (0) (negative), 0.3–1 (1) (low), 1–15 (2 + 3) (moderate-high), > 15 (4) (very high)] was 79,4% (Fig. 1). Good agreement was found for all relevant shared egg, cow’s milk, peanut, codfish/ black tiger prawn, soy and wheat components. Between overlapping nut components (hazelnut, walnut, cashew- and brazil nut) overall class agreement was moderate with 68%, however in line with previous studies [1–4] overall agreement (both positive / both negative) was good with 83%.

*Differences* between overlapping storage proteins in ALEX and ISAC were found for hazelnut (Cor a 14) and walnut (Jug r 1) (both high sensitization in ALEX while negative in ISAC) in patient 2 and patient 5 (no challenge tests for hazelnut and walnut performed; so clinical relevance unknown). Verification of this difference was performed using a singleplex sIgE test (ImmunoCap) on the same serum sample. In patient 2 no Cor a 14 sensitization conform ISAC and in patient 5 Jug r 1 sensitization conform ALEX was confirmed to be correct. One clinically irrelevant difference was found in patient 1 who tolerates small amounts of soy. In this patient, Gly m 5 sIgE was negative in ALEX while moderately elevated in ISAC.

*Additional positive sensitizations* for many allergens were found by ALEX (Table 1) without resulting in additional food allergy diagnosis (not diagnosed by ISAC) in these ten patients so far. In all ten patients multiple additional sensitizations, mainly for allergen extracts, with unknown clinical relevance were found (Table 1). In patient 2 moderate sensitization for buckwheat component (storage protein, Fag e 2) and high sensitization for whole allergen buckwheat extract was found by ALEX, with no sensitization in ISAC for buckwheat component. Because of unexplained anaphylaxis in this patient a buckwheat food challenge was performed, however this was negative in agreement with ISAC. In addition, in seven patients ALEX revealed sensitizations for buckwheat extract with unknown relevance in five of them. Furthermore, many sensitizations (low – very high) all with unknown clinical relevance were found by ALEX for a.o. extracts of chickpeas (n = 2), lentils (n = 2), fenugreek (n = 4), poppy seeds (n = 8), lupin (n = 5), storage mites including Acarus siro (n = 7), cricket, mealworm and grasshopper (n = 5) and components of beer yeast (n = 3) and honeybee (n = 1). A food challenge with poppy seed was performed in patient 2, showing unexplained anaphylaxis and high sensitization for poppy seed found by ALEX, but was negative. Patient 8 with dust mite and seafood allergy (sensitized for tropomyosin) showed very high sensitizations for cricket, mealworm and grasshopper extracts in ALEX (possibly tropomyosin cross-sensitizations). Therefore he was advised to avoid those insects in his diet, however no food challenge tests with these insects were performed so clinical relevance remained unknown.

*Negative sensitizations* (on allergen components/extracts not present in ISAC) were found by ALEX to eleven seafood species in patient 5 and 8 with fish allergy so both patients were allowed to introduce seafood (Table 1). In both patients ALEX showed no sensitization for tuna whole allergen extract while sensitization for the parvalbumin component (Thu a 1) was very high. Remarkably, in all other fishes the sensitization for parvalbumin was considerably higher than whole fish allergen extracts. Very low or negative sensitization was found for specific nuts in four out of ten patients with a nut allergy (Table 1). This could help clinicians to decide together with their patients to perform a food challenge with these specific nut(s) in case of a desired wish for introduction. In line with this, in two out of four patients with a known allergy for legumes, negative or very low sensitization was found for specific legumes which might be worth challenging (Table 1). Negative sensitization for banana extract was found by ALEX in patient 1. He avoids banana because of oral allergy and abdominal symptoms and a positive skin pricktest for banana six years ago.

This is the first study comparing ISAC and ALEX multiplex arrays in atopic children by relating their sensitization results to clinical data. Like previous studies [1–4] good overall agreement was found for egg, cow’s milk, peanut, codfish/prawn, soy and wheat components. Although this is a pilot study with limited patient numbers, based on our study the sensitivity / specificity of nut components in both systems and tuna whole allergen extract in ALEX needs to be further investigated. It is important to keep in mind that ALEX and ISAC report different units (kU/L vs ISU-E) and ranges so clinicians will need some experience translating them into their daily clinical practice. Comparing both tests ALEX provides more sIgE results (including whole allergen extracts and components) for nuts, cereals, seeds, legumes, fish and seafood, fruits, vegetables, meats and spices. In general, multiplex arrays could help clinicians to study cross-sensitization patterns in individual patients, however for this purpose allergen components are more informative than allergen extracts. In this study no additional food allergies were identified by ALEX compared to ISAC, however in case of negative sensitization results clinicians could more safely optimize and personalize dietary advice (Table 1). This study showed multiple sensitization results found by ALEX with unknown clinical relevance, which could potentially increase clinical complexity and costs by an increase in further evaluation (food challenge tests) to establish relevancy of sensitizations. Pediatric allergists should be aware of this disadvantage of using ALEX, even more in young children with (severe) eczema who have not introduced all sorts of food yet. In theory a more extended testing platform could be interesting in patients with unexplained anaphylaxis, however this was not studied in the present pilot study.

This study was limited by patient numbers, however based on the results of this study, future studies should investigate the impact of additional positive sensitizations found by ALEX on clinical decision making and healthcare cost in severe atopic children.

## Data Availability

The data that support the findings of this study are available on request from the corresponding author [LS]. The data are not publicly available due to participant privacy.

## References

[1] Heffler E, Puggioni F, Peveri S, Montagni M, Canonica GW, Melioli G (2018). Extended IgE profile based on an allergen macroarray: a novel tool for precision medicine in allergy diagnosis. World Allergy Organ J.

[2] Bojcukova J, Vlas T, Forstenlechner P, Panzner P (2019). Comparison of two multiplex arrays in the diagnostics of allergy. Clin Transl Allergy.

[3] Scala E, Caprini E, Albeni D, Meneguzzi G, Buzzulini F, Cecchi L, Villalta D, Asero R (2021). A qualitative and quantitative comparison of IgE antibody profiles with two multiplex platforms for component-resolved diagnostics in allergic patients. Clin Exp Allergy.

[4] Quan PL, Sabaté-Brescó M, D'Amelio CM, Pascal M, García BE, Gastaminza G, Blanca-López N, Alvarado MI, Fernández J, Moya C, Bartra J, Ferrer M, Goikoetxea MJ (2022). Validation of a commercial allergen microarray platform for specific immunoglobulin E detection of respiratory and plant food allergens. Ann Allergy Asthma Immunol.

[5] Hoang JA, Celik A, Lupinek C, Valenta R, Duan L, Dai R, Brydges MG, Dubeau A, Lépine C, Wong S, Alexanian-Farr M, Magder A, Subbarao P, Upton JEM, Schmidthaler K, Szépfalusi Z, Ramani A, Eiwegger T (2021). Modeling the conversion between specific IgE test platforms for nut allergens in children and adolescents. Allergy.

